# Sustained engagement with a digital youth mental health platform: A mixed-methods study

**DOI:** 10.1016/j.invent.2025.100899

**Published:** 2025-12-12

**Authors:** Lee Valentine, Jennifer Nicholas, Rory Sorenson, Nicola A. Chen, Carla McEnery, Shona Louis, Shane Cross, Shaminka N. Mangelsdorf, Shaunagh O'Sullivan, Thomas W. Wren, Sandra Bucci, John Gleeson, Sarah Bendall, Mario Alvarez-Jimenez

**Affiliations:** a Orygen, The National Centre of Excellence in Youth Mental Health, Melbourne, Australia; b Centre for Youth Mental Health, The University of Melbourne, Melbourne, Australia; c Goulburn Valley Public Health Unit, Goulburn Valley Health, Victoria, Australia; d Division of Psychology and Mental Health, School of Health Sciences, Manchester Academic Health Sciences Centre, The University of Manchester, Manchester, UK; e Greater Manchester Mental Health NHS Foundation Trust, Manchester, UK; f Healthy Brain and Mind Research Centre, School of Behavioural and Health Sciences, Faculty of Health Sciences, Australian Catholic University, Melbourne, Australia

**Keywords:** Digital mental health, Digital intervention, Mental health, Youth mental health, Young people, Engagement, Motivation

## Abstract

**Background:**

Digital mental health interventions offer a scalable means of expanding access to youth mental health care. However, their capacity to achieve population-level impact is limited by persistently low and varied user engagement. A more nuanced understanding of experiential factors associated with sustained engagement is needed to inform the design of digital interventions that are both clinically effective and engaging to young people.

**Objective:**

To identify experiential factors associated with sustained engagement with a large-scale, real-world digital youth mental health platform implemented across Australian youth mental health services.

**Methods:**

A convergent mixed-methods approach integrated platform usage data and qualitative interviews with young people aged 16–25 years (mean = 19; *n* = 36) classified as either high or low engagers. The sample comprised 53 % female, 31 % male, and 8 % trans and/or gender diverse; 3 % identified as First Nations, and 19 % as culturally and linguistically diverse. Most participants lived in metropolitan areas (82 %), with 8 % from regional locations. Interview transcripts were thematically coded for experiential features of engagement. Code presence was quantified and tested for association with engagement level (high vs low) using Fisher's exact test. Themes were then iteratively refined, with explicit consideration of experiential patterns significantly associated with engagement.

**Results:**

Two experiential factors were significantly associated with sustained engagement: (1) relational experiences, including normalisation and validation (*P* = 0.038) and, community and belonging (*P* = 0.01); and (2) the practical application of therapeutic insights in everyday life (P = 0.03). Low motivation was commonly reported as a barrier but did not significantly differentiate high from low engagers (*P* = 0.467).

**Conclusions:**

This mixed methods study offers preliminary insights into experiential factors associated with engagement with a large-scale digital mental health intervention for young people. Indications suggest two experiential pathways to sustained use: relational experiences characterised by feeling understood, normalisation, and belonging, and functional utility reflected in the practical application of therapeutic strategies in daily life. Challenges reported by low-engaged young people, including cognitive overload, low motivation, and limited reminder prompting from the intervention, highlight opportunities for adaptive, low-effort re-engagement strategies. As digital mental health services continue to scale, understanding engagement as an emotional, social, and practical process may help support more sustained and inclusive participation.

## Highlights

• High and low engagers reported qualitatively different experiences of use.
• Relational experiences and practical utility were associated with higher engagement.
• Young people described passive use as meaningful.
• Low motivation and cognitive overload contributed to disengagement.

## 1 Introduction

Mental ill-health is one of the most significant health challenges facing young people globally (McGorry et al., 2024). It now accounts for a substantial proportion of the total disease burden in this age group, estimated at around 45 % for those aged 10–24 years (Gore et al., 2011; McGorry et al., 2022). Evidence indicates that both the prevalence and severity of mental ill-health among young people are steadily increasing (McGorry et al., 2024). This escalating burden highlights the urgent need for scalable, evidence-based youth-focused responses at the population level.

However, the growing demand for mental health care has not been met with a proportional increase in access to appropriate and timely mental health care (McGorry, 2022; Lehtimaki et al., 2021). Many young people face substantial barriers to treatment, related to long wait times (MacDonald et al., 2021), workforce shortages (Crocker et al., 2023), stigma (Sheikhan et al., 2023), and low mental health literacy (Beckman et al., 2023). These structural and systematic barriers often result in delayed diagnoses and unmet need (McGorry, 2022; Lehtimaki et al., 2021). Early intervention during this period is critical to mitigating distress and improving longer term outcomes (McGorry et al., 2022), yet young people remain the most affected yet least likely to receive adequate mental health treatment (McGorry et al., 2022; Burns and Birrell, 2014).

Digital mental health interventions (DMHIs) offer a way to address this need, and recent Australian state and federal reforms have prioritised the digital transformation of mental health services (Government Department of Health A, 2020). As a scalable, flexible, and potentially cost-effective means of reaching underserved populations, DMHIs are increasingly positioned as a key strategy to address the growing gap between mental health need and service availability.

Despite the promise of DMHIs, one of the most persistent implementation challenges is low engagement (Fleming et al., 2018; de Beurs et al., 2017; Lipschitz et al., 2022; Torous et al., 2025). Many users never initiate use after being offered or consenting to a digital intervention, with some studies reporting that up to half of participants fail to download the app (Torous et al., 2025). Among those who do engage initially, sustained use is uncommon, drop-off is rapid, and full program completion is rare. For instance, research has shown that nearly 50 % of users allocated to widely used mental health apps, such as Headspace or Smiling Mind, discontinued use within ten days (Torous et al., 2025). These engagement difficulties are consistently observed across different user groups and settings, posing a significant barrier to the broader population-level impact of digital mental health care (Fleming et al., 2018; de Beurs et al., 2017; Lipschitz et al., 2022; Torous et al., 2025).

A recent meta-analysis by Gan et al. (2021) found that higher levels of engagement with DMHIs were modestly but significantly associated with improvements in mental health outcomes across a range of populations, conditions, and intervention types. While engagement alone does not guarantee therapeutic benefit, a minimum threshold of engagement, defined here in behavioural terms such as the amount, frequency, duration, and depth of interaction with a digital intervention, is likely necessary to achieve meaningful clinical outcomes. However, the precise threshold required remains poorly understood in the digital health field. These findings underscore the need to move beyond quantifying behavioural engagement alone, toward a deeper understanding of the contextual and individual experitential factors that shape engagement variability in real-world settings.

Emerging evidence suggests that not only the amount, but also the type or quality of engagement may influence clinical outcomes in digital mental health. In a study of young people using the Moderated Online Social Therapy (MOST) platform, a youth mental health intervention that combines structured therapeutic content, a moderated online community and clinician, vocational, and peer support, O'Sullivan et al. (2022) identified distinct engagement patterns linked to differential clinical outcomes. Young people who engaged with both the therapeutic content and the social community component showed greater clinical improvement, whereas those who engaged solely with the social community did not. A subsequent analysis by O'Sullivan et al. (2023) demonstrated that social community engagement can serve as a catalyst for therapeutic engagement, suggesting that while interaction with the social community alone may not be sufficient to drive clinical change, it can play a critical role in facilitating broader, outcome relevant engagement with the platform.

Engagement is likely shaped by a range of interacting factors (Zhang et al., 2019). Reported challenges include technical difficulties, poor user experience, lack of personalisation, intervention complexity, privacy concerns, digital poverty, and low intrinsic motivation (Borghouts et al., 2021; Eisner et al., 2025; Valentine et al., 2022). Conversely, facilitators such as social connection, increased autonomy, co-design (Thabrew et al., 2018), and access to human support are associated with enhanced experiences of engagement (Lehtimaki et al., 2021; O'Sullivan et al., 2022; Borghouts et al., 2021; Valentine et al., 2024). Collectively, these findings suggest that engaging digital interventions must address multiple, interacting factors rather than relying on a singular design approach.

However, even high levels of engagement do not necessarily translate into clinical improvement (Yardley et al., 2016). Behavioural change, the mechanism through which psychological interventions typically confer benefit, requires ongoing support to be maintained over time (Lipschitz et al., 2023). Without such reinforcement, treatment effects often diminish over time (Pilling et al., 2020). This challenge is not unique to any one intervention; studies across a range of DMHIs, including those targeting early psychosis (Hassan et al., 2023), have reported variability in user engagement and corresponding outcomes. These findings underscore the importance of designing digital interventions that not only foster initial engagement but also support sustained behaviour change to achieve lasting outcomes (Yardley et al., 2016).

Persuasive design and user experience (UX) strategies have been explored as mechanisms to improve engagement with DMHIs, yet findings remain inconsistent. A recent systematic review and meta-analysis found no significant relationship between the type, number, or combination of persuasive design principles and engagement with mental health apps (Valentine et al., 2025). Similarly, while UX is frequently cited as a key driver of engagement (Majumder, 2025), evidence in this area varies. For instance, Kaveladze et al. (2022) found that although highly rated apps had better user experience scores, these ratings did not predict long-term engagement. Likewise, in a study assessing technology acceptance and intention to use DMHIs among 248 young people recruited via social media (41 % female; most aged over 18; 47 % had previously used a DMHI; 61 % reported a mental health need), Sawrikar and Mote (2022) found no association between perceived ease of use and willingness to engage. These findings suggest that improving UX alone may be insufficient in ensuring sustained engagement.

A more comprehensive understanding of engagement requires attention to how young people experience and make meaning of DMHIs. While existing frameworks enable quantitative analysis of potential engagement factors, they may oversimplify the complexity of user experiences. In contrast, qualitative studies adopting a bottom-up approach can offer rich, contextualised insights into the lived experience that shape engagement (Valentine et al., 2020a, Valentine et al., 2020b; Garrido et al., 2022). However, as purely qualitative methods do not lend themselves to statistical generalisability, it remains challenging to establish clear, measurable associations between engagement drivers and outcomes, and to draw actionable conclusions that can inform targeted intervention design at scale. To address this gap, the study will employ a convergent mixed-methods design to examine experiential factors that distinguish high- and low-engagement during the first 12 weeks of participation in a large scale, real world digital mental health service for young people.

## 2 Method

### 2.1 Study design and setting

This mixed-methods study was embedded within the national implementation of Moderated Online Social Therapy (MOST), a multi-component digital mental health platform integrated into routine care across public youth mental health services in Australia. Supported through state and federal funding, a group of implementation facilitators work with services to locally adapt an implementation plan to support the use of MOST in the service. Included services span community-based primary mental healthcare and specialist services. This large-scale rollout provides an unprecedented real-world context for examining digital engagement at scale. At the time of data collection, the platform had been implemented in 258 services across Victoria, New South Wales, and Queensland. As part of standard care, the platform was routinely offered to young people referred to these services, including those on waitlists.

MOST (https://most.org.au) combines interactive therapeutic content, a moderated peer-support community, and access to clinical, peer and, vocational support (Alvarez-Jimenez et al., 2019, Alvarez-Jimenez et al., 2020, Alvarez-Jimenez et al., 2021; Cross et al., 2023; Engel et al., 2024; Rice et al., 2018). Accessible via web or smart phone app, the platform was developed in consultation with young people and other key stakeholders. It enables both structured, evidence-based modules and self-paced engagement. MOST can function as a standalone intervention or as part of blended care during waitlist, active treatment, or discharge phases, with coordination between MOST clinicians and primary care teams (Cross et al., 2025).

### 2.2 Participants & eligibility

The eligible sample comprised young people aged 15–26 years who were receiving routine care through Australian youth mental health services and had been offered the MOST digital intervention as part of standard service delivery. These were real-world service users who had completed a 12-week course of the MOST intervention and whose platform usage placed them in either the upper or lower quartiles of engagement. At the time the engagement data were analysed to define these quartiles, 4407 young people had completed the onboarding process for the MOST platform.

Participants for the qualitative component were selected from this clinical population using a stratified sampling approach designed to compare experiences between those with minimal versus sustained engagement. Eligibility was limited to young people who had not opted out of research follow-up during onboarding procedures.

### 2.3 Engagement classification and sampling strategy

Engagement with the MOST platform was defined by the number of *active days*, measured as the total number of unique days a participant logged onto the platform at least once during the first 12-week intervention period. Thresholds were derived empirically based on the distribution across the full user base, with high engagement defined as ≥12 active days (upper quartile), and low engagement as 1–4 active days (lower quartile).

The sampling strategy incorporated purposive recruitment to ensure meaningful representation from groups historically underrepresented in research yet disproportionately affected by mental ill-health, including First Nations young people, culturally and linguistically diverse young people, and LGBTIQA+ young people, and young men. This approach recognised that marginalised groups often face distinct mental-health challenges arising from systemic discrimination and exclusion (Gee and Ford, 2011), and that these perspectives are critical for informing equitable and culturally responsive digital mental health care. Including these voices enhances the contextual relevance of the findings and supports the design of digital mental-health interventions that better meet diverse user needs and can work to avoid perpetuating existing inequities common in traditional service models (Hui et al., 2021).

### 2.4 Recruitment

A target sample of 40 participants, 20 from each of the high- and low-engagement groups, was determined based on pragmatic considerations and power estimates sufficient to support meaningful mixed-methods analysis. In total, 452 young people met eligibility criteria for the high-engagement group (upper quartile, ≥12 active days), and 259 were eligible for the low-engagement group (lower quartile, 1–4 active days).

In the high-engagement group, all 452 eligible young people were contacted once via text message. Recruitment concluded once 22 high-engagement participants had completed interviews.

In the low-engagement group, all 259 eligible young people were contacted via text message, followed by two reminder messages to improve response rates. Fourteen participants from this group completed interviews.

### 2.5 Data collection

Semi-structured interviews were conducted between March and August 2023 via Zoom by authors RS and LV, following completion of electronic informed consent via REDCap, hosted by the University of Melbourne (Harris et al., 2009, Harris et al., 2019). Interviews were guided by an interview schedule designed to explore participants' experiences of the MOST platform, including their initial expectations, responses to onboarding, patterns of use, and experience of components (e.g. therapy content, peer and clinician support, the online community) (Appendix 1). Interviews were video recorded to facilitate transcription and analysis. Recordings were transcribed verbatim by a third-party service (https://rev.com/), and all audio, video, and transcript files were securely stored on a password-protected server. Identifying information, including PDFs of the completed electronic consent forms, was stored separately to maintain confidentiality. Access to recordings and transcripts was limited to the immediate research team responsible for conducting the analysis.

### 2.6 Ethical considerations

The study received approval from the Royal Melbourne Hospital Human Research Ethics Committee (HREC/83853/MH-2022). Written informed consent was obtained from all participants using REDCap.

Participants were provided with a plain-language statement of the study's aims, procedures and potential risks and benefits. They were informed that they could skip questions, take a break, or withdraw at any time without consequence. Participants were also advised that they could withdraw their interview data from the study for up to one week following participation. All participants received a digital copy of the participation information and consent form and were reimbursed AUD $30 via bank transfer or e-gift card. All data were de-identified and stored on a secure, password protected server.

### 2.7 Data analysis

#### 2.7.1 Mixed methods approach

A mixed-methods approach was used to examine the relationship between participant engagement level with the MOST platform and experience of use. This approach was informed by Usher-Smith et al. (2017) and combined inductive thematic analysis of qualitative interview data (Braun and Clarke, 2006) with quantitative platform usage metrics. The qualitative component involved inductive coding of interview transcripts, while the quantitative component applied Fisher's Exact Test to examine if endorsement of specific codes differed significantly between high- and low-engagement groups.

Authors RS and LV independently coded the first interview transcript in Dovetail (https://dovetail.com/). Through iterative discussion and reflection, the coders developed a preliminary coding framework. This process was repeated for an additional five transcripts, with each coder working independently, followed by joint review and refinement of the coding framework. Once consensus was reached, RS applied the finalised framework to the remaining 30 transcripts, with ongoing consultation and reflexive practice with LV to ensure consistency throughout the coding process.

Many codes were identified during this process. To identify experiential factors with the greatest potential influence on engagement, authors RS, LV, and senior author SB selected the most frequently endorsed codes, those reported by a substantial proportion of participants, as candidate themes for integration with usage data. These codes were chosen based on their prevalence and their conceptual relevance to engagement, allowing the mixed-methods analysis to focus on experiences most likely to shape patterns of use.

Integration of qualitative and quantitative data was conducted using a mixed methods matrix (see Table 3), in which each row represented an individual participant (*n* = 36), along with their engagement level (high vs low). For example, endorsement of the code “sense of belonging” was compared across participants with high versus low engagement to assess whether this experience was significantly associated with sustained platform use.

To quantitatively test these relationships, Fisher's Exact Test was applied to each code to determine whether endorsement rates differed significantly by engagement level. This test was selected because it is well-suited to small samples and unequal group sizes, as was the case in this study. Alpha was set at 0.05, and no adjustment for multiple comparisons was applied because the analyses were exploratory. Codes that showed statistically significant associations with engagement level and those that were prevalent but not significantly associated were explored in greater depth through thematic analysis. This included identifying subthemes that captured nuanced differences in how key experiences, such as connection, motivation, or normalisation, were described by participants across levels of engagement.

## 3 Results

The sample included 36 young people (14 low engagers, 22 high engagers), with a mean age of 19 years (range 15–26). Both the high and low engagement groups had a recruitment success rate of 5 %. Gender identities were diverse, with 52.8 % identifying as female, 30.5 % as male, and 8.3 % as trans and gender diverse. Additionally, 2.8 % identified as First Nations and 19 % as culturally and linguistically diverse. Most participants were located in metropolitan areas (82 %), with 8 % living in regional areas. Participant characteristics are summarised in Table 1. Interviews averaged 31.9 min in duration (range from 17.0 to 67.2 min).

**Table 1:** Participant characteristics by engagement group.

Characteristic	Category	High engagement (*n* = 22)	Low engagement (*n* = 14)
Gender	Male (cisgender)	4 (18.2 %)	3 (21.4 %)
	Male (transgender)	4 (18.2 %)	–
	Female (cisgender)	8 (36.4 %)	9 (64.3 %)
	Female (transgender)	1 (4.5 %)	–
	Female and non-binary	1 (4.5 %)	–
	Female and genderqueer	1 (4.5 %)	–
	Female and unsure	1 (4.5 %)	–
	Gender diverse	1 (4.5 %)	–
	Agender	1 (4.5 %)	–
	Not answered	–	2 (14.3 %)
Age	15–17	6 (25.0 %)	4 (28.6 %)
	18–20	7 (36.1 %)	6 (42.8 %)
	21–23	8 (33.3 %)	4 (28.6 %)
	24–26	1 (5.6 %)	–
First nations	Yes	1 (2.8 %)	–
	No	33 (91.6 %)	12 (91.6 %)
	Not answered	2 (5.6 %)	2 (14.3 %)
Culturally & linguistically diverse	Yes	7 (19.4 %)	3 (21.4 %)
	No	27 (75.0 %)	9 (64.3 %)
	Not answered	2 (5.6 %)	2 (14.3 %)
Geographic location	Metro	33 (91.7 %)	12 (85.7 %)
	Regional	3 (8.3 %)	2 (14.3 %)

### 3.1 Qualitative themes identified

The analysis of young people's experiences with the MOST platform identified six themes: (1) normalisation and validation; (2) community and belonging; (3) the practical application of therapeutic insights into everyday life; (4) in-the-moment support, (5) flexibility and autonomy in self-guided mental health support, and (6) low motivation. Themes are explored in detail below.

### 3.2 Associations between themes and level of platform engagement

Fisher's exact tested identified significant associations between several themes and level of engagement with the MOST platform (see Table 2). Higher engagement was significantly associated with experiences of normalisation and validation (*P* = 0.038), community and belonging (*P* = 0.01), and the practical application of therapeutic insights into everyday life (P = 0.03). In contrast, in-the-moment support (*P* = 0.733), flexibility and autonomy in self-guided mental health support (*P* = 0.142), and low motivation (*P* = 0.467) were not significantly associated with level of engagement.

**Table 2:** Fisher's exact test results comparing experiential themes across high and low engagement groups.

Theme	Endorsed by whole sample, n (%)	Endorsed in low engagement group, n (%)	Endorsed in high engagement group, n (%)	*p*-Value
Normalisation and validation	19 (53 %)	4 (29 %)	15 (68 %)	0.038
Community and belonging	16 (44 %)	3 (21 %)	13 (59 %)	0.01
The practical application of therapeutic insights into everyday life	14 (39 %)	2 (14 %)	12 (55 %)	0.03
In-the-moment support	18 (50 %)	6 (48 %)	12 (55 %)	0.733
Flexibility and autonomy in self-guided support	11 (31 %)	2 (14 %)	9 (41 %)	0.142
Low motivation	11 (31 %)	3 (21 %)	8 (36 %)	0.467

**Table 3:** Mixed-methods matrix ordered according to level of engagement (low/high), where dots indicate the presence of that theme within the qualitative interview data.

Engagement level	Participant						
	ID	Normalisation and validation	Community and belonging	The practical application of therapeutic insights into everyday life	In-the-moment support	Flexibility and autonomy in self-guided mental health support	Low motivation
Low	4	●	●	●	●		
	5	●					●
	9						
	10	●	●	●	●		
	13				●		
	18				●		
	22					●	
	24		●				
	25					●	
	26				●		●
	28				●		
	30						
	31	●					
	32						●
High	1	●		●			●
	2	●		●		●	●
	3	●		●		●	●
	6	●	●	●	●	●	
	7	●	●		●	●	●
	8						
	11	●	●	●	●	●	
	12	●	●	●	●		
	14	●	●	●		●	●
	15		●	●			●
	16			●			
	17	●	●		●		
	19	●			●		
	20	●	●	●		●	●
	21	●			●		●
	23			●	●		
	27	●	●		●	●	
	29		●	●	●		
	33		●				
	34	●	●		●		
	35		●		●		
	36	●				●	

### 3.3 Qualitative themes associated with high engagement

#### 3.3.1 Theme 1: normalisation and validation

Young people in the high-engagement group were significantly more likely than those in the low-engagement group to describe the intervention as normalising and validating of their mental health experiences (*p* = 0.038). Specifically, 68 % (*n* = 15) of highly engaged participants reported feeling reassured by seeing their experiences reflected in their peers' posts and comments on the platform, compared to 29 % (*n* = 4) of those with low engagement. Many described a sense of relief in realising they were “*not the only person going through this* [P6]” and recognising their own experiences in others helped reduce feelings of isolation.

This theme reflected an internal, largely introspective experience. Young people described a cognitive and emotional shift triggered by recognising their own struggles in others' stories, even without interacting with others young people in the community directly.

*“I guess it just made me, like, feel less alone in it. Like even if I didn't actively participate, it's just like, ‘Oh, yeah. These people are going through the same thing.”*

[YP20]

For some, reading about how others experienced and navigated similar difficulties prompted a shift in perspective, supporting participants to reframe their own challenges as part of a broader, shared experience rather than personal failings. This recognition functioned primarily as a form of self-reappraisal, offering comfort and validation:

*“It was really reassuring knowing that, okay, this is not all in my head...this is a very normal thing. Millions of people experience it as well.”*

[YP5]

In some cases, this experience not only reduced feelings of isolation but also prompted participants to reflect on and share their own coping strategies with community.

*“Just scrolling through and reading about that specific issue. If it's about depression, then you see how different people discuss how they battled through it or why they went through it, then you can share, “Yeah, I kind of had the same experience too. I didn't feel like the happiest in that time, but then I did these few things to help me, and I think they might work for you.“ It felt like you weren't alone. There's actually other people around your age that go through the same things as you.”*

[YP11]

The intervention's community component supports both passive engagement (e.g., reading and observing) and active participation (e.g., posting, commenting, using emoji reactions), thereby accommodating varying levels of comfort and readiness among participants. For many, mere exposure to other young people's stories, without any direct interpersonal exchange, was enough to produce a sense of being understood.

*“It was less of talking that helped me; it was more of seeing how other people managed similar feelings... That's what helped me. I let it out, and now I know that someone out there is going through or has gone through the same feelings and can possibly help or just listen, I guess.”*

[YP19]

These peer narratives provided more than emotional validation, they also served as a form of behavioural modelling. Observing how others navigated similar mental health challenges provided a framework for interpreting and managing their own struggles. This observational support was a key mechanism through which young people felt understood and supported, even in the absence of direct communication.

High-engagement participants were more likely to report that seeing peers openly discuss their experiences and coping strategies was both reassuring and instructive. The social nature of this exposure was central to its impact; however, it did not depend on direct interaction or reciprocal contact. Instead, participants described a form of social observation, in which simply witnessing others' disclosures helped them make sense of their own experiences. This theme therefore centred on feeling personally normalised and understood through the presence of others, rather than on forming interpersonal connections. In this way, the peer-driven environment enabled a reinterpretation of distress, where individual struggles became recognisable as part of a broader, collective experience.

#### 3.3.2 Theme 2: community and belonging

The perception of the intervention as a supportive community in which young people belonged was a key differentiator between young people with high and low engagement. Those in the high engagement group were significantly more likely to describe the platform as a space in which they felt acknowledged, supported, and understood by peers (59 %, *n* = 12) compared to those with low engagement (14 %, *n* = 2; *p* = 0.0142). This sense of social cohesion was not dependent on direct, one-to-one interactions. Rather, it was experienced as embedded within the platform's design and community ethos, which fostered normalisation, understanding, and mutual support among young people experiencing mental health challenges. Whereas Theme 1 centred on internal recognition and self-reassurance, this theme captured a more outward-facing, relational sense of being part of a collective.

Participants described how even brief interactions, such as receiving an emoji in response to a community post or comment, was experienced as meaningful gestures of care. As one participant described:

“*Getting a love heart, and then caring emojis, makes me feel really happy, like people actually care for me, even though I don't know them.*”

[P34]

For some, the community filled a critical gap, particularly for those who found it difficult or undesirable to share their mental health struggles with friends or famliy, or who lacked relationships in which such conversations felt possible. As participant 7 explained:

*“I don't really have a lot of friends I can reach out to. So, the fact that I can do it in a safe space with, like, the peer workers moderating the posts and stuff, I think that was good. ‘Cause sometimes I just, I don't really wanna tell my friends. And I could see a lot of the peer workers really providing that, like, support and keeping the community, like, a positive and safe environment.”*

*[P7]*

The platform was described not only as a resource during period of distress, but also as a source of community and support that young people could return to over time. For many in the higher engaged group, it functioned as a reliable safe space to share life updates, during both everyday moments and times of heightened need. As one participant explained,

*“If something significant is happening, I might jump on there and make a post...it's a welcoming environment to talk about just about anything...it's really good to just, just slap life updates in there and then you know, just talk to people about it.”*

[P29]

The capacity for flexible, self-directed engagement helped reinforce the platform's role as a stable and accessible source of support over time.

Importantly, the community is also shaped by conversations that extended beyond mental health, such as discussions about hobbies, creative interests, and everyday experiences. These lighter interactions, such as sharing posts about D&D [Dungeons and Dragons], comics, and creative writing, as well as light-hearted questions like *“What's your favourite animal? Or what's your favourite song right now? [P15]”* played a role in creating a space that felt approachable and aesthetically pleasing. Participants shared personal moments, such as photos of their pets, contributing to a broader tapestry of personal identity and community. As one participant explained,

“*The idea of it… all coming together on common ground. These are people who are looking for advice and solidarity… it makes it less scary.”*

*[P35]*

Taken together, these experiences suggest that young people's engagement was shaped not only by the platform's therapeutic content but also by the sense of co-presence created through ongoing interactions, shared activities, and the visibility of others navigating similar challenges. This atmosphere of being alongside others, without requiring the pressure of direct conversation or reciprocal exchange, helped the platform feel like a place where young people could return, and feel part of a broader peer context. For many, it was this sustained sense of being “with” others in a safe, responsive space that fostered feelings of community and belonging.

#### 3.3.3 Theme 3: the practical application of therapeutic insights into everyday life

Young people in the high-engagement group were significantly more likely than those in the low-engagement group to report gaining practical insights through the intervention that supported their mental health in everyday life (58 %, *n* = 12 vs. 14 %, *n* = 2; *p* = 0.0334). These insights were drawn from various components of the intervention, including psychoeducational modules, interactions with online clinicians and peer workers, and peer-generated community content. Participants frequently described these learnings as concrete, actionable, and directly relevant to their day-to-day experiences.

Participants described how the intervention introduced key mental health concepts in ways that helped them make sense of their thoughts, emotions, and behaviours by “j*ust like getting a better awareness of it [P20].”* In offering language and frameworks for interpreting negative or overwhelming experiencing, the intervention enabled participants to reframe challenging experiences as understandable and thus more manageable. This process often alleviated confusion and fostered a sense of control.

*“When I first started, it made me do a quiz to figure out what the main thing I was dealing with was, and I put in the panic attack and anxiety thing. And so, they designed a course that took me through what it was and things that I could learn about undoing certain thought patterns... one of them that I remember is something about rumination where it just taught me what that was... just took me through all that in a really easy understandable way... It made it feel like it was a normal, explainable thing, whereas when you're experiencing it, you feel like this is so random and weird, I don't know how to explain what's going on right now. But the way they designed those sessions, I guess, made it seem very logical—like this is fine. If this happens, it's cool, you can deal with it.”*

*[YP14]*

Here, the structured nature of the intervention enabled the participant to make sense of anxiety and rumination as recognisable and explainable phenomena, rather than confusing or anomalous experiences. What had previously felt like a strange, inexplicable reaction was reframed as a common and manageable response. This accessibility of information and acknowledgement as a common experience offered reassurance and clarity, transforming uncertainty into understanding and fostering a greater sense of control.

These learning moments were not confined to the intervention. Participants described applying what they learned to real-world situations, such as recognising and managing anxiety in unfamiliar settings or re-evaluating interpersonal expectations with regards to making friends. One participant described how learning that anxiety in unfamiliar situations is a typical response, and this knowledge helped them feel more at ease in social situations.

*“When I'm feeling anxious about a new situation, having someone say it's perfectly natural to be anxious… where you've never been before… was incredibly helpful.”*

*[YP23]*

Another participant reflected on how a module on relationship-building helped shift their expectations around forming connections with others. By outlining that meaningful relationships take time to develop, the intervention reduced feelings of pressure but also supported behaviour change outside of the intervention, encouraging greater patience and more realistic approaches to fostering relationships.

*“I think that kind of struck a chord with me... I would meet someone and feel connected, but it wasn't really happening… being aware that it just takes time with people to build connection made me more patient.”*

*[YP2]*

These findings suggest that DMHIs can serve not only as support tools but also as mechanisms for cognitive restructuring, equipping young people with conceptual and practical frameworks to navigate mental health challenges more effectively. By fostering both self-awareness and applied learning, such interventions may enhance resilience and promote engagement with mental health strategies in everyday life, extending the interventions impact beyond the digital context.

### 3.4 Qualitative themes not associated with levels of engagement

#### 3.4.1 Theme 4: in-the-moment support

Using the platform during episodes of heightened anxiety, stress or emotional overwhelm was reported by participants across both high- and low-engagement groups, with no statistically significant difference between groups (*P* = 0.733). Overall, 50 % (*n* = 18) of participants described accessing the platform in such moments, suggesting that the intervention functioned as a readily available source of support irrespective of engagement level.

Young people frequently described turning to the platform as a self-initiated coping mechanism during difficult moments. The intervention was experienced not only as a source of distraction but also provided structured techniques for emotional regulation and peer validation and advice. Several participants described engaging with the community to process distress, finding comfort in peer narratives that mirrored their own struggles and finding practical strategies from others.

*“I think the last time I used it, I might've had a panic attack. I have to do something to distract myself, which is usually a game, I think I jumped onto MOST. I was going to talk to a peer worker, but I moved from a peer worker to the community and decided to read what's happening. I think I was reading a post about someone having a panic attack or their anxiety and it was like, “Oh my God, I totally understand that. “And I'm pretty sure they put down tips on how they dealt with it. So, I tried it.”*

*[P19]*

This ability to access information and coping strategies provided immediate emotional relief for some young people. As one participant described, the platform was particularly valuable during period of acute distress when other supports were unavailable:

*“Sometimes I used to get panic attacks like in the middle of the night. So I found it helpful when it was like 3:00 a.m., 4:00 a.m., everyone's asleep in the house and I don't wanna bother people. And I shouldn't call the ambulance once again. So, it was helpful in those areas, and it's accessible.”*

*[P17]*

Notably, six participants from the high-engagement group described using the platform specifically at night, often during periods of heightened vulnerability, restless, rumination or sleep disturbance. In these moments, the platform functioned as a form of connection and support when other avenues were unavailable.

*“Times of confusion, distress that sort of thing. Like, where I need to wind down and be in a community where there's people going through similar things, and where there's tools that can help me sort of de-escalate and get through the rest of the night I guess.”*

*[P27]*

These accounts suggestion that for subset of young people, the platform was not simply a tool for structured therapeutic engagement, but a psychologically available and emotionally responsive intervenient, particularly valued during late-night hours when emotional distress could feel more isolating. In this context, the platform's combination of community and practical support appeared to offer a unique an accessible form of care.

#### 3.4.2 Theme 5: flexibility and autonomy in self-guided mental health support

The self-guided nature of the intervention was commonly described as flexible, accessible, and supportive of young people's autonomy in managing their mental health. This theme was endorsed across both engagement groups, with 32 % (*n* = 7) of participants in the high engagement group and 14 % (*n* = 2) in the low engagement group, with no statistical difference between groups (*p* = 0.142).

Young people described that the intervention's flexibility was particularly valuable in the context of busy or unpredictable schedules, enabling them to integrate mental health support into everyday life with greater ease.

*“I could take it with me wherever. I could use it whenever. And I could sort of go through activities and that sort of thing at my own pace. Go back to them... Yeah.”*

*[P27]*

This was particularly relevant for those balancing work, study, or other competing demands, where synchronous support (e.g. in person or telehealth) was less practical.

*“I liked that you could do it any time, before bed… Whereas telehealth was through, like, Zoommeetings. And I still had to schedule my time around it. Whereas MOST is more, yeah, self-paced.”*

*[P2]*

The absence of fixed timelines or attendance requirements was perceived by some as reducing pressure and contributing to a greater sense of control. In this way, the platform was less likely to become another source of stress. Participant 22 explained,

*“There was no like, oh, if it's not done well like, this time like, you don't have access to it, or like, you're gonna be cut off... I found it, because there wasn't like, a time constraint, it was a lot more relaxing.”*

*[P22]*

Young people also emphasised the value of being able to choose, change and revisit therapeutic content based on their changing needs. Participant 3 described switching between modules,

“*I've started, like, half of them... Find Your Calm, Find Your Confidence, Improve Your Mood... just bouncing back in between depending on which one I feel like doing or which one I feel is relevant to my mood of the night*.”

For a number of participants, MOST served as their primary source of mental health support. The ability to access help independently and without clinician direction was seen as both empowering and convenient.

*“MOST is my main source of mental health information and support... because you can do the journeys without having a clinician assign them to you... it's there for me when I need to and I don't have to wait if I need to access it.”*

*[P11]*

Together, these experiences illustrate how the self-directed nature of MOST enabled some young people to access support in a way that felt adaptable, non-burdensome, and integrated into their everyday lives, offering both therapeutic support and a sense of control.

#### 3.4.3 Theme 6: low motivation

Difficulty with motivation, particularly during periods of low mood or anxiety, was reported by participants across both the high and low engagement groups, with no statistically significant difference between them (*P* = 0.467). In total, 31 % (*n* = 11) of participants described struggling to initiate or sustain engagement with the platform, suggesting that motivational challenges may be a common barrier irrespective of overall usage level.

For some, use of the platform was described as inconsistent and largely reactive, at times occurring after being reminded in face-to-face appointments:

*“Very intermittently, there was no real* consistency *to it...I had my sessions with my in-person clinician...and then I'm sort of reminded like, ‘Oh, yeah, you know, MOST things, I might go home and like do a couple of modules or something.”*

*[P1]*

Others indicated that engagement was often contingent on prompting from a MOST clinician. This form of contact served as both motivational scaffolding and a source of accountability;

*“Those check-ins, like motivated me to continue trying.... I think when I was having panic attacks, I... used to do a lot of breathing exercises. But at one point I got really lazy. But when I had the MOST clinician checking in with me, yeah, it definitely helped me.”*

*[P7]*

Participants described how depressive symptoms, and general inertia made it difficult to engage in self-guided support

*“I just didn't really care. Like, I was just happy lying in bed and being sad.... I feel like I need an appointment time, and need the, like, oh, you've got a cancellation fee if you don't rock up.”*

*[P26]*

For some, anxiety and social withdrawal further impeded engagement, particularly with the platform's interactive features, echoing patterns of common in in-person interactions:

*“Just with.... depression, anxiety, I was a bit isolated... Going and just talking to new people... that was quite scary... going online to that as well was a bit, like, intimidating.”*

*[P4]*

## 4 Discussion

### 4.1 Principal findings

This study aimed to identify experiential factors associated with young people's engagement with a digital youth mental health intervention implemented at scale in youth mental health services across Australia. Using a convergent mixed-methods approach, we integrated platform usage data with in-depth qualitative interviews to examine how and why young people remained engaged, or disengaged, over time. Three qualitative themes related to high engagement were identified: (1) normalisation and validation, (2) community and belonging, and (3) the practical application of therapeutic insights in everyday life. An additional three themes, (1) in-the-moment support, (2) flexibility and autonomy in self-guided mental health support, and (3) low motivation, were not significantly associated with engagement level but offer valuable implications for intervention design.

Findings highlight two preliminary experiential factors related to sustained engagement: relational experiences, such as feeling understood, normalisation of mental health challenges, and a sense of belonging, and perceived usefulness, reflected in the ability to apply therapeutic content in everyday life. Given the modest sample size and exploratory nature of this work, these results should be interpreted as early indications rather than definitive evidence of causal mechanisms. Nonetheless, they point to two emerging pathways to engagement: one grounded in social and relational processes, and the other in the practical utility of therapeutic content. Each pathway carries distinct implications for optimising platform design and for refining theoretical models of digital mental health engagement.

Young people in the high-engagement group commonly described a sense of normalisation and belonging derived from observing others sharing similar struggles. These experiences reportedly helped reduce feelings of isolation and provided reassurance, even in the absence of direct interaction with others. Low-effort social interaction, such as emoji reactions or brief comments, were described as meaningful and contributed to a sense of felt community. Although these patterns are suggestive, they should be viewed as exploratory, as causation cannot be inferred, and further research is required to determine whether and how these social experiences contribute to sustained engagement. However, these findings do align with literature on social presence and the role of relational factors in digital mental health engagement (Lehtimaki et al., 2021; Yardley et al., 2016; Baumel et al., 2019; Baumel, 2022) and reinforce the role of relatedness, a core tenant of self-determination theory, in sustaining motivation and participation (Deci and Ryan, 2008).

Interestingly, these interactions also reflected a reciprocal process. Participants described both being supported and supporting others, speaking to the mutuality central to the Intentional Peer Support (IPS) framework (MacNeil and Mead, 2003). Storytelling was reported to help shape, or reshape, internal narratives around mental health by allowing participants to reflect on their own experiences in relation to others'. This reciprocity, where both ‘poster’ and ‘reader’ benefit, reinforces the need for digital interventions to move beyond static peer content and toward co-created, lived-experience narratives that foster collective meaning making and community. While the value of digital peer support is increasingly recognised, rigorous evaluation of these models remains limited (Rayland and Andrews, 2023), highlighting the importance of continued research in this area.

A substantial proportion of participating highly engaged young people who participated in the interviews identified as transgender and/or gender diverse (41 %). This may partly reflect the greater appeal of digital mental health platforms to LGBTQIA+ youth, for whom online spaces often provide safety, anonymity, and freedom from the marginalisation experienced in offline settings. Recent evidence indicates that most LGBTQIA+ young people turn to online communities to connect with others when in-person support is limited, and 78 % report feeling more able to express their full selves online (The Trevor Project, 2025). Systematic reviews further suggest that digital interventions can alleviate mental health symptoms among LGBTQIA+ youth (Liu et al., 2023), while social media and online environments serve as essential safe spaces for identity exploration and support when offline contexts are “unwelcoming” (Bacaj et al., 2024). This dynamic may have contributed to the higher representation of transgender and/or gender diverse participants in the high-engagement group and their greater willingness to share their experiences, reflecting both their familiarity with digital environments and their motivation to engage with inclusive, affirming services.

The importance of recognising passive engagement was also identified. Many participants described benefitting from simply reading the posts of others, even if they did not post themselves or actively complete therapy modules. This form of passive participation is frequently missed by conventional behavioural engagement metrics such as number of posts or modules completed, yet may still represent meaningful interaction with both peer communities and therapeutic content. Indeed, in this study, passive engagement of this kind may have contributed to an increased ‘active day’ count, resulting in some young people being classified in the high engagement group despite minimal visible activity. This highlights why engagement in DMHIs is difficult to quantify, a young person may gain substantial value through passive use, but this may not be adequately reflected in available usage metrics or traditional notions of ‘dose’. Designing for passive engagement, through written, audio, and video content, curated peer contributions, and varied modes of reflection, may help better capture and support the full spectrum of meaningful use.

Alongside social connection, the ability to apply therapeutic strategies to daily life was identified as a key driver of sustained engagement. Participants described using cognitive and behavioural learnings from the platform to manage emotions, navigate relationships and structure daily activities. These experiences appeared to reinforce the perceived value and utility of the platform, possibly creating self-reinforcing cycles in which tangible benefits may have increased motivation to return to the platform. Given the exploratory design however, this interpretation should be regarded as hypothetical rather than conclusive but does align with evidence from digital behaviour change literature, where skill generalisation and perceived efficacy are associated with greater engagement and improved outcomes (Kelders et al., 2020).

Taken together, these findings suggest that engaging DMHIs may benefit from design that supports both social connection and real-world applicability. While the former addresses the need for normalisation and belonging, the latter enhances perceived usefulness and supports behavioural change, both of which appear to contribute to sustained engagement. Social features may also serve as gateways to therapeutic content. Prior research has shown that engagement with peer networks may increase engagement with structured therapeutic components, indirectly supporting clinical improvement (O'Sullivan et al., 2022). Future iterations of MOST and similar platforms may benefit from intentionally designing moderated peer spaces that are psychologically safe, support lived experience storytelling and are embedded with clear pathways to therapeutic content and tools.

Low engagement was commonly associated with low motivation, anxiety and general overwhelm. These findings are consistent with the Digital Cumulative Complexity Model (DiCuCoM) (Cross and Alvarez-Jimenez, 2024), which proposes that when the burden of engagement exceeds a person's cognitive or emotional capacity, disengagement becomes more likely. For some participants, the action of remembering to log into the platform, without the aid of reminders or prompts, was experienced as an insurmountable task. This highlights the need for digital interventions to minimise cognitive load and incorporate low-effort re-engagement strategies, such as timely, personalised notifications. Similar findings were reported by Valentine et al. (2024) in a qualitative study of a digital intervention for young people with repetitive negative thinking. Participants described difficulty engaging with the app during episodes of distress, citing low energy, cognitive fatigue, and concerns that confronting negative thoughts might exacerbate their emotional state. Although the app offered potential therapeutic benefit, the perceived effort required to engage was, for some, prohibitively high. Taken together, findings from both studies underscore the importance of designing digital interventions that are sensitive to users' cognitive and emotional capacity, particularly during periods of heightened distress.

By integrating usage metrics and experiential data, this study contributes to a more nuanced understanding of engagement with DMHIs. While quantitative metrics provide useful indicators of use, they often fail to capture the psychological and emotional processes that underlie sustained engagement. Qualitative insights, conversely, offer rich accounts of these processes but are limited in generalisability and quantifiability. Through a convergent mixed-methods design, we identified statistically significant experiential factors associated with high engagement, while also exploring the mechanisms by which these experiences may influence use. This integrative approach enables more targeted and translatable engagement strategies, supporting the design of DMHIs that are engaging and responsive to the lived realities of young people.

### 4.2 Limitations

Several limitations should be acknowledged. First, interviewers were not explicitly blinded to participants' engagement level at the time of interview, which may have introduced bias during data collection. However, engagement level was not linked to transcripts during analysis, and coders were unaware of participants' engagement level, reducing the risk of confirmation bias in interpreting qualitative data. Reflexive qualitative practices, including independent coding and comparison, iterative peer discussion, and regular supervision, were also employed to help mitigate bias. Nonetheless, such strategies cannot fully eliminate the influence of researcher subjectivity.

Second, recruiting young people in the low-engagement group was challenging, an expected difficulty in real-world service contexts where lower engagers are typically less reachable and less likely to opt into research. This resulted in a smaller number of participants in the low-engagement group. Because participation relied on young people opting in, it is possible that those who chose to take part, particularly among high engagers, may have felt more positive toward the platform than young people who did not participate in interviews. Importantly, however, we still observed statistically significant differences in the experiences described by high and low-engagement participants, suggesting that some distinctions were detectable despite the potential for positive priming among participants.

Although uneven group sizes and opt-in recruitment may have influenced the balance of perspectives captured and reduced power to detect subtler differences, these findings should be interpreted cautiously. The imbalance between groups could mean that themes not reaching statistical significance may reflect limited power rather than an absence of difference. As such, these patterns point to areas that warrant further investigation in larger, more representative samples.

Finally, the study design does not allow the directionality of associations between experiential factors and engagement to be established. It remains unclear whether high-engagement participants reported greater feelings of normalisation, belonging, and opportunities to apply therapeutic content because they spent more time on the platform, or whether early experiences of these processes preceded and facilitated sustained engagement. Conversely, low-engagement participants may have had fewer opportunities to encounter elements of the platform that support connection or skill use. Accordingly, the experiential differences observed here should be interpreted as correlational. Longitudinal or experimental approaches will be necessary to determine whether these experiential processes actively contribute to sustained engagement or primarily reflect varying levels of exposure.

Taken together, these limitations indicate that the findings provide an initial foundation for understanding engagement-related experiences, but further research is required to establish their generalisability and causal significance.

## 5 Conclusion

This study offers preliminary insights into the experiential factors associated with engagement with a large-scale, real-world digital mental health intervention for young people. By integrating platform usage data with qualitative interviews, we identified early indications of two pathways that may underpin sustained engagement: relational experiences, such as feeling understood, normalisation of mental health challenges, and a sense of belonging, and functional utility, reflected in the practical application of therapeutic strategies in everyday life. These observations align with theories that emphasise young people's basic psychological needs for relatedness and competence, and they provide support for approaches that embed socially meaningful and skill-building opportunities within digital interventions (Deci and Ryan, 2008). Further research, however, is needed to test, refine, and validate these emerging propositions (Yardley et al., 2016; Baumel et al., 2019).

Insights from low-engagement users highlighted additional challenges, including cognitive overload, low motivation, and how a lack of reminders can limit use. These experiences point to the value of adaptive, low-effort re-engagement strategies, such as personalised notifications, that accommodate fluctuating mood and motivation.

Importantly, passive forms of engagement, such as reading peer stories without active contribution, were experienced as meaningful for some participants yet these forms of use are often excluded from conventional metrics, possibly due to difficulty in measuring them These observations suggest a the need for a broader conceptualisation of what constitutes therapeutic interaction within digital platforms.

As digital mental health services continue to scale, addressing variability in engagement is important for enhancing both clinical effectiveness and equitable access. A more nuanced understanding of engagement, beyond frequency or duration, may help capture its emotional, social, and practical dimensions. Designing interventions that incorporate peer support, accommodate varied modes of participation, and minimise complexity could help promote sustained and inclusive use, particularly for those facing greater barriers to engagement.

## Abbreviations

DMHIs: Digital mental health interventions
MOST: Moderated Online Social Therapy
UX: User Experience
HREC: Hospital Human Research Ethics Committee
IPS: Intentional Peer Support
DiCuCoM: Digital Cumulative Complexity Model
LGBTIQA+: Lesbian, Gay, Bisexual, Transgender, Intersex, Queer, Asexual, Plus

## Author contributions

LV, SBendall, and JN conceptualised the study. LV, JN, and SBendall developed the study methodology. NC conceptualised the analytic approach to understanding engagement and conducted the analysis of usage data, including classification of users into high and low engagement groups. LV and RS conducted the qualitative interviews. LV, RS, SBendall, and SL contributed to the mixed-methods analysis. LV wrote the original draft of the manuscript. All authors (JN, NC, CM, SL, SC, SNM, SO, TW, SBendall, JG, SBucci, and MAJ) contributed to the review and editing of the manuscript. All authors approved the final version.

## Funding

Professor Bendall reports financial support was provided by Australian 10.13039/501100000925National Health and Medical Research Council (2026634). Professor Alvarez-Jimenez was supported by an investigator grant (APP1177235) from the National Health and Medical Research Council and a Dame Kate Campbell Fellowship from the University of Melbourne. Dr. Nicholas is supported by a National Health and Medical Research Council Emerging Leader Fellowship (ID 2009782). Professor Bucci acknowledges support for this work from a UK National Institute for Health and Care Research (10.13039/501100000272NIHR) research professorship (grant NIHR300794) and the NIHR Manchester Biomedical Research Center (grant NIHR203308). The views expressed are those of the authors and not necessarily those of the NIHR or the Department of Health and Social Care.

## Declaration of competing interest

The authors declare that they have no known competing financial interests or personal relationships that could have appeared to influence the work reported in this paper.

## Data Availability

Access to the data is available upon request and will be considered in accordance with ethical and institutional guidelines.

## Appendix A Supplementary data

Appendix 1: Interview schedule Image 1
