# Supplementary material for: Sustained engagement with a digital youth mental health platform: A mixed-methods study
Source: Internet Interv. 2025 Dec 12;43:100899. doi: 10.1016/j.invent.2025.100899 (PMC12811672; doi:10.1016/j.invent.2025.100899)
Supplement: Supplementary file 1 — Appendix 1: Interview schedule [file mmc1.docx]

**Appendix 1: Interview Schedule**

| Question | Prompt |
| --- | --- |
| When you first heard about MOST, what made you think, *“Okay, sure, I’ll give it a go”* |  |
| What were you hoping MOST might help you with? | Thinking back now, do you feel it met those expectations?  Is there anything that could have made getting started easier or more helpful? |
| Do you remember what it was like to sign up or get started on MOST? |  |
| How did you find the onboarding process, for example, the steps you took to set things up or get introduced to the platform? | Do you remember what it felt like? |
| When and how would you usually use MOST? (e.g., time of day, how often, what prompted you to log in?) |  |
| What was it like to actually use MOST? | How did you find the therapy journeys or activities?  What was the online community like? (e.g., *Talk It Out* discussions, social network)  Did you ever connect with peer workers or clinicians through MOST? What was that like?  Did you have any contact with the Careers and Study team? If so, how was that experience? |
| Have you ever used MOST alongside face-to-face support from a clinician? | **(If yes)** What was that experience like?  **(If no)** Move on. |
| Can you think of a specific time when you used MOST? Could you walk me through it? |  |
| Looking back overall, what stands out to you most about your experience with MOST? |  |
| Is there anything you think could be improved or added to make it more useful or engaging for young people? |  |
| Is there anything else you’d like to share about your experience with MOST? |  |
